# Italian Orthopaedic and Traumatology Society (SIOT) position statement on the non-surgical management of knee osteoarthritis

**DOI:** 10.1186/s10195-023-00729-z

**Published:** 2023-09-07

**Authors:** Elisa Pesare, Giovanni Vicenti, Elisaveta Kon, Massimo Berruto, Roberto Caporali, Biagio Moretti, Pietro S. Randelli

**Affiliations:** 1 Orthopaedic & Trauma Unit, Department of Basic Medical Sciences, Neuroscience and Sense Organs, School of Medicine, University of Bari Aldo Moro, AOU Consorziale “Policlinico”, Piazza Giulio Cesare 11, 70100 Bari, Italy; 2 Biomechanics and Technology Innovation Laboratory, II Orthopaedic and Traumatologic Clinic, Rizzoli Orthopaedic Institute, Bologna, Italy; 3 Nano-Biotechnology Laboratory, Rizzoli Orthopaedic Institute, Bologna, Italy; 4 ASST Centro Specialistico Ortopedico Traumatologico Gaetano Pini, CTO, Milan, Italy; 5 Department of Clinical Sciences and Community Health, University of Milan, and IRCCS S Matteo Foundation, Pavia, Italy; 6 Laboratory of Applied Biomechanics, Department of Biomedical Sciences for Health, Università Degli Studi Di Milano, Via Mangiagalli 31, 20133 Milan, Italy; 7 U.O.C. 1° Clinica Ortopedica, ASST Centro Specialistico Ortopedico Traumatologico Gaetano Pini-CTO, Piazza Cardinal Ferrari 1, 20122 Milan, Italy; 8 Research Center for Adult and Pediatric Rheumatic Diseases (RECAP-RD), Department of Biomedical Sciences for Health, Università Degli Studi Di Milano, Via Mangiagalli 31, 20133 Milan, Italy

**Keywords:** Osteoarthritis, Knee OA, SIOT position statement, Non-surgical management

## Abstract

**Background:**

Knee osteoarthritis (OA) is a chronic disease associated with a severe impact on quality of life. However, unfortunately, there are no evidence-based guidelines for the non-surgical management of this disease. While recognising the gap between scientific evidence and clinical practice, this position statement aims to present recommendations for the non-surgical management of knee OA, considering the available evidence and the clinical knowledge of experienced surgeons. The overall goal is to offer an evidenced-based expert opinion, aiding clinicians in the management of knee OA while considering the condition, values, needs and preferences of individual patients.

**Methods:**

The study design for this position statement involved a preliminary search of PubMed, Google Scholar, Medline and Cochrane databases for literature spanning the period between January 2021 and April 2023, followed by screening of relevant articles (systematic reviews and meta-analyses). A Società Italiana Ortopedia e Traumatologia (SIOT) multidisciplinary task force (composed of four orthopaedic surgeons and a rheumatologist) subsequently formulated the recommendations.

**Results:**

Evidence-based recommendations for the non-surgical management of knee OA were developed, covering assessment, general approach, patient information and education, lifestyle changes and physical therapy, walking aids, balneotherapy, transcutaneous electrical nerve stimulation, pulsed electromagnetic field therapy, pharmacological interventions and injections.

**Conclusions:**

For non-surgical management of knee OA, the recommended first step is to bring about lifestyle changes, particularly management of body weight combined with physical exercise and/or hydrotherapy. For acute symptoms, non-steroidal anti-inflammatory drugs (NSAIDs), topic or oral, can be used. Opioids can only be used as third-line pharmacological treatment. Glucosamine and chondroitin are also suggested as chronic pharmacological treatment. Regarding intra-articular infiltrative therapy, the use of hyaluronic acid is recommended in cases of chronic knee OA [platelet-rich plasma (PRP) as second line), in the absence of active acute disease, while the use of intra-articular injections of cortisone is effective and preferred for severe acute symptoms.

## Highlights

1. NSAIDs (topical or oral formulations) are a better choice for acute symptoms, compared with acetaminophen in knee osteoarthritis non-surgical treatment.
2. SIOT recommend opioid use only while patients are waiting for surgical treatment, if NSAIDs are ineffective against pain.
3. Considering intra-articular infiltrative therapy, the use of hyaluronic acid is recommended in cases of chronic knee OA in the absence of active acute disease, while the use of intra-articular injections of cortisone is effective and preferred for severe acute symptoms.
4. The use of growth factor injections and/or PRP in symptomatic knee OA is only favoured in highly specialised centres

## Introduction

Osteoarthritis (OA) is the most common form of arthritis and a major cause of disability [1]. The most common site of OA is the knee joint, with an estimated overall prevalence in the general adult population of 24% [2]. The frequency of this condition is bound to increase further due to population ageing.

Recommendations for the management of knee OA have been published by several different scientific authorities including, amongst others, the Osteoarthritis Research Society International (OARSI) [3], the American College of Rheumatology (ACR) [4], the American Academy of Orthopedic Surgeons (AAOS) [5, 6], the European League Against Rheumatism (EULAR) [2] and the European Society for Clinical and Economic Aspects of Osteoporosis and Osteoarthritis (ESCEO) [1]. We have collated recommendations from these sources and combined them with the results of an extensive literature search, using our own expert knowledge to produce a set of evidence-based recommendations for the non-surgical management of this condition.

## Material and methods

A working group of five Società Italiana Ortopedia e Traumatologia (SIOT) members was established, consisting of four orthopaedic surgeons and a rheumatologist with extensive experience in the treatment of knee OA and the analysis and interpretation of related evidence. One member of the task force (EP) collected the literature, searching entries in PubMed, Google Scholar, Medline and Cochrane databases dated between January 2011 and August 2021. Keywords for the search included ‘osteoarthritis’, ‘knee OA’, ‘guidelines’, clinical practice’, ‘non-surgical management’ and ‘conservative treatment’, and the results were limited to ‘humans’, ‘randomised controlled trial’, ‘meta-analysis’, ‘review’ and ‘systematic review’. Inclusion and exclusion decisions were based on group consensus. A second researcher (GV) independently verified the number of articles identified to avoid potential discrepancies. Study characteristics and data were extracted onto a Microsoft Excel spreadsheet.

The following data were extracted for each study: first author, title, design of the study and year of publication. Initially, titles and abstracts of all records were reviewed. Only full-text articles written in English were included, and several articles were excluded after this preliminary review process. Full-text copies of the studies were then obtained and assessed by the authors.

The Preferred Reporting Items for Systematic Reviews and Meta-analyses (PRISMA) guidelines [7] were followed.

**Fig. 1:**
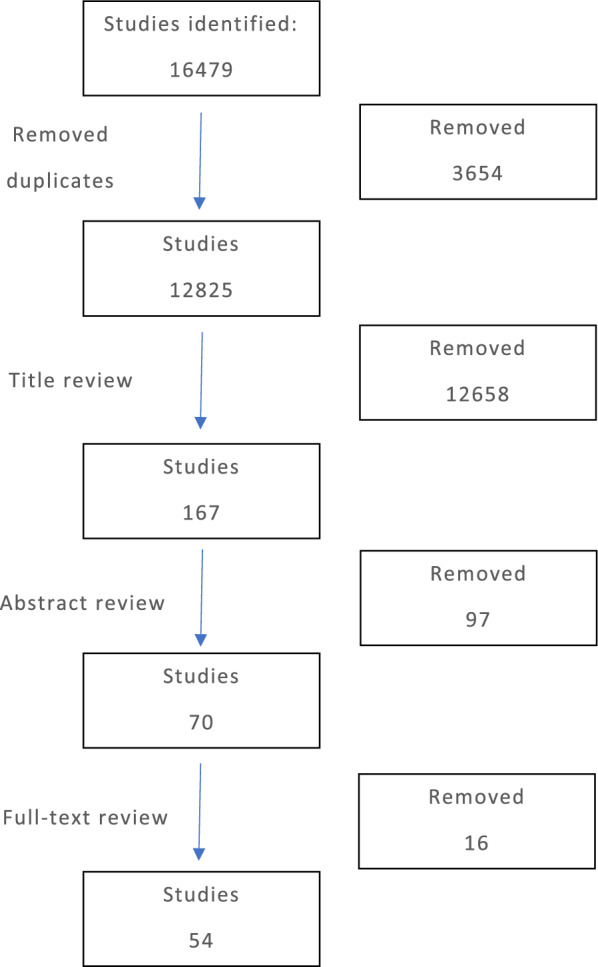
Flowchart of study selection

The material was presented to the task force in an initial meeting. A total of 16,479 articles were identified in the following databases: PubMed, Cochrane, Medline and Google Scholar. Overall, 3654 duplicates were removed. After inspection of the titles and abstracts and applying the inclusion criteria, a total of 30 studies were reviewed further (Fig. 1).

In subsequent meetings, a schematic chart of conservative treatment recommendations for knee OA was agreed by task force members.

The consensus of the working group was based on both evidence from the literature and expert opinion.

By electronic communication, it was possible to draft the manuscript, sharing corrections and suggestions from individual members with the rest of the team.

Among the several available recommendations for the management of knee OA, those from the Osteoarthritis Research Society International (OARSI) [3], the American College of Rheumatology (ACR) [4], the American Academy of Orthopedic Surgeons (AAOS) [5, 6], the European League Against Rheumatism (EULAR) [2] and the European Society for Clinical and Economic Aspects of Osteoporosis and Osteoarthritis (ESCEO) [8] were selected for examination.

## Results

### Lifestyle and physical therapy

#### Weight management

This represents one of core treatments for knee OA, in combination with exercise and self-management programmes. SIOT strongly recommended core treatment in early onset OA and in mild/moderate OA, as well as in severe cases. Weight loss is considered to be effective in those who are overweight [body mass index (BMI) ≥ 25 kg/m^2^) or obese (BMI ≥ 30 kg/m^2^). Specifically, loss of ≥ 5% of body weight can be associated with changes in clinical and functional outcomes [4].

#### Self-management and education

SIOT consider self-management and education one of the core treatments together with weigh management and exercises. Structured patient education programmes aim to inform patients about their condition and the available treatment strategies, to reduce the likelihood of disease progression and severity. Awareness regarding OA aetiology, risk factors (especially if modifiable), expected prognosis and therapeutic strategies can help to reduce misunderstandings and mistakes in patients (for example, the misconception that physical exercise can be harmful to the joints). Education of family members can also be useful. Self-management and education are also strongly recommended by the OARSI [9], EULAR [2], AAOS [5, 6] and ESCEO [8].

#### Balneotherapy/spa therapy

Balneotherapy represents a conservative treatment that may have beneficial effects on pain and stiffness, with a tolerable economic profile [10]. It consists of the use of thermal waters that are therapeutically active by virtue of mineral composition, mud and natural gas. In numerous papers, balneotherapy is described as a treatment with favourable results [11, 12]. SIOT moderately recommend the use in mild OA.

#### Canes, walking sticks, crutches, walkers

Depending on the severity of the disease and the needs of each patient, these devices can aid walking, significantly reducing the load on the lower limbs, improving stability and assisting movement. The risk of falls also appears to be reduced [4, 11]. Walking assist devices are strongly recommended in patients with symptomatic knee OA.

#### Exercise (land and water based)

For individuals with knee OA, the types of exercises performed on land include muscle strengthening, aerobic stretching and neuromuscular balance exercises, and more. [13] However, most importantly, any proposed programme should be based on patient needs [8, 9]. Water offers natural resistance, which helps strengthen muscles [14, 15]; evidence shows that exercise in water provides improvements in pain and quality of life in people who are unable to perform land-based exercise due to pain. SIOT consider land and/or aquatic exercise one of core treatments together with weight loss and self-management and education.

#### Pulsed electromagnetic field therapy (PEMT).

Evidence that PEMT significantly improves pain and function in people with knee OA is low in quality due to the short-term nature of the follow-ups described in the literature [16]. Thus, further studies with long-term follow-ups should be performed. Cardiovascular deficiencies, blood sugar levels disorders, blood coagulation diseases and anti-coagulant therapies are relative contraindications in PEMF treatment [5, 17]. There is a lack of consensus in literature about duration, frequency, and intensity of PEMF therapy sessions [18].

Nevertheless, PEMT has proved therapeutically effective for bone- and cartilage-related pathologies and can be used to reduce pain and stiffness [19].

PEMT may be used to improve pain and/or function in patients with mild knee OA [20]; therefore, the SIOT recommendation is moderate**.**

#### Bisphosphonate

Bisphosphonates are anti-resorptive agents (currently used in the treatment of osteoporosis). They represent a potential candidate for osteoarthritis therapy [21, 22]. Results from evidences using bisphosphonates in OA have been encouraging but controversial: some studies suggest neridronate is effective in OA treatment [23], while others contend that clodronate could play a role as a disease-modifying drug. OARSI is weakly favourable to risedronato due to the few studies in literature supporting its application as a reducer of the marker of cartilage degradation (CTX-II) which may contribute to slow the radiological progression of OA, particularly in patients who are not overweight [24, 25]. On the other side, AAOS and ACR do not recommend their use [5, 26]. Limitations of the studies included differences in the bisphosphonate analysed, the dose and the route of administration [27]. Future studies are needed: SIOT recommendation on their use is inconclusive.

#### Oxygen–ozone therapy (O_3_ therapy)

Ozone is known for its anti-inflammation effect and its work on cellular metabolism [28]. In knee OA, O_3_ therapy is described as a safe approach with encouraging effects [29] with respect to pain control and functional recovery in the short-to-middle term [30], with an almost null adverse event rate [31] especially in combination with other treatments [28].It is contraindicated in patients with a significant deficit of G-6PD, in pregnancy, in case of hyperthyroidism, thrombocytopenia and serious cardio-vascular instability [32]. SIOT recommendation to its use in knee OA is limited.

#### Transcutaneous electrical nerve stimulation (TENS)

TENS uses a low-voltage electrical current delivered through electrodes attached to the patient’s skin to stimulate peripheral nerve activity (neuromodulation) [33–35]. TENS can be generally delivered at two different dosing, high frequency (50e100 Hz) and low frequency (2e10 Hz): the use of TENS is not recommended in people with pacemakers and women who are pregnant should not apply TENS in the abdominal or pelvic regions [5]. The literature on this is highly heterogeneous, and the available clinical trials are characterised by short follow-up periods. Thus, SIOT consider the available evidence insufficient to recommend this procedure [16].

### First-line pharmacological treatment (management of acute symptoms)

#### Acetaminophen (or paracetamol)

This is generally used to treat mild-to-moderate pain [36]. It is weakly recommended as an initial pharmacological approach in the presumption of its overall safety [8, 37]. However, while the OARSI recommends against its use in both the short and long term, the ESCEO and ACR make a weak recommendation for its use in the short term, and the AAOS strongly recommends its use [5, 5, 38]. SIOT moderately recommend acetaminophen at doses no greater than 3 g/day in mild/moderate OA if not contraindicated (in cases of hypersensitivity to acetaminophen, severe hepatic impairment or severe active liver disease) [5].

Topical non-steroidal anti-inflammatory drugs (NSAIDs).

Topical use of NSAIDs is recommended as first-line treatment, particularly in patients with comorbidities, owing to their proven efficacy and low risk of gastrointestinal (GI), cardiovascular or renal adverse events (OARSI, ACR, ESCEO, AAOS). Topical NSAIDs can be applied as gel, cream, spray or patch formulations to the skin of the affected area [4, 8]. SIOT strongly recommend their use in patients with comorbidities with symptomatic knee OA.

### Second-line pharmacological treatment (management of persistent symptoms)

#### Oral NSAIDs

Oral NSAIDs are strongly recommended for use in knee OA. They are more effective than acetaminophen in most people (OARSI, ACR, ESCEO, AAOS). The potential harms of NSAIDs are well known and include GI, renal and cardiovascular adverse effects. Elderly people, who are at higher risk of OA, are also at higher risk of experiencing these side effects. Therefore, these drugs should be used with caution in elderly patients [39].

SIOT recommends the use of non-selective NSAIDs, preferably with the addition of a proton pump inhibitor (PPI) or selective COX-2 inhibitors [40]. For individuals with GI comorbidities, selective COX-2 inhibitors and non-selective NSAIDs in combination with a PPI are conditionally recommended due to their benefits regarding pain. Doses should be as low as possible, and NSAID treatment should be continued for as short period as possible**.**

### Third-line pharmacological treatment (management of refractory symptoms)

#### Duloxetine (anti-depressant drug)

The analgesic efficacy of duloxetine in central pain is presumably due to its influence on the descending pathways of pain inhibition, it is contraindicated in patients with liver failure or severe renal dysfunction, uncontrolled angle-closure glaucoma and concurrent or recent therapy with monoamine oxidase (MAO).

The OARSI [3], ACR [4] and ESCEO [37, 41] recommend this drug in patients with knee OA and widespread pain and/or depression. The AAOS does not provide any recommendations on its use [5, 5] Evidence suggests that duloxetine presents with some tolerability issues, being associated with adverse events such as nausea, dry mouth, drowsiness, fatigue, constipation, decreased appetite and hyperhidrosis [4].

SIOT conditionally recommended duloxetine as the last line of pharmacological therapy in patients who are candidate for surgery treatment.

#### Opioids (oral)

Opioids can be appropriate for use if other therapies are ineffective or if feasible surgical options are lacking. The OARSI does not recommend opioid use in patients who have persistent symptoms over a long period of time, due to the risk for development of tolerance [38]. Therefore, they should only be used for short periods and as a last resort [3, 5, 6, 26] before considering switching to surgical treatment. SIOT recommend the use of oral opioids in short-term therapy in patients with refractory OA who are awaiting planned surgical treatment [11].

#### Opioids (transdermal)

In opioid-tolerant individuals, SIOT encourages the use of transdermal patch, rather than oral formulations. The indications are the same as for oral opioids: patients on the waiting list for surgery, with refractory symptoms. This formulation has delayed onset of effects but prolonged duration of action [26]. Application to the skin avoids first-pass hepatic metabolism, increasing bioavailability and limiting fluctuations in plasma concentration. However, the OARSI recommendations [3] discourage the use of opioids with transdermal patch formulation following poorly documented clinical benefits and the high risk of addiction and adverse events [11].

#### Diacerein and IL1-inhibition.

These drugs are a group of agents able to block the activity of a proinflammatory cytokine, IL-1, which is believed to play a role in inducing the degradation of cartilage matrix through the upregulation of proteolytic enzymes [38]. The ESCEO working group underline that the benefits of diacerein are more than its risks and confirms that it can be an option for knee OA treatment [42].

Diacerein should be avoided in patients with a propensity for diarrhoea and could be useful in patients with contraindications to NSAIDs [43].

However, SIOT do not recommend the use, due to its cost and limited benefits.

### Chronic pharmacological treatments

#### Glucosamine and chondroitin

Glucosamine and chondroitin are strongly recommended against for knee OA, even though they are commonly used in clinical practice. To date, the available studies are burdened by several discrepancies and biases. The OARSI and ACR, strongly recommend against the use of glucosamine and chondroitin, AOOS [5, 5] consider this therapy helpful in improving functional outcomes in patients with mild/moderate knee OA, and conversely, ESCEO [37] guidelines recommend these treatments as first-line therapy.

The SIOT recommendation to use glucosamine and chondroitin is weak, and is limited for individuals with chronic knee osteoarthritis [16]. According to data sheets, adults should take these supplements orally twice a year, for almost 2 months each day at doses of 1200 mg of glucosamine and chondroitin.

Allergies to shellfish, asthma or patients using warfarin or diabetes drugs are considered conditions that do not preclude the use of glucosamine, but individuals with these conditions should be closely monitored for any potential side effects including bloating, nausea, diarrhea and constipation [43].

### Intra-articular injection treatments: first line (acute symptoms)

#### Corticosteroids (intra-articular injection)

Intra-articular glucocorticoid injections are strongly recommended for patients with knee OA to relieve pain in the short term (2–4 weeks). However, clinicians should be cautious about the potential damage of repeated and long-term use (> 6 weeks) [44]. The AAOS provide a moderate recommendation for use, focusing on the risks associated with repeated injections, while other societies such as the OARSI, ACR and ESCEO recommend short-term treatment. SIOT encourage the use in patients with acute episodes of disease exacerbation once a week for not more than 3 weeks [45], even though literature did not generally provide insights into a recommended schedule for repeated injections. The repeated use of intra-articular glucocorticoids, particularly in mild-to-moderate stages of knee OA severity, may have negative effects, according to recent studies [46].

Absolute contraindications to the use of corticosteroid injections are infection, sepsis and bacteremia, and joint instability. Juxta-articular osteoporosis (because of the risk of subchondral osteonecrosis and weakening of the joint structures), coagulopathy and long-term therapy are relative contraindications [47].

### Intra-articular injection treatments: first line (chronic therapy)

#### Hyaluronic acid

Intra-articular hyaluronic acid (IAHA) shows a more favourable long-term safety profile [40] than intra-articular corticosteroids. However, according to the OARSI, ACR and AAOS, there is little evidence regarding effectiveness [3, 5, 26]. IAHA are ideal for patients who do not have adequate pain relief from oral medications (NSAID, acetaminophen), exercise and physical therapy, or patients with existing renal or gastrointestinal intolerance for NSAIDs [48].

There is no absolute contraindication of intra-articular injection of HA other than acute inflammation in the joint cavity, although the drug effect may be reduced in the following cases. It is prohibited for use in diseases such as extensive bone edema, bone fissure or stress necrosis on magnetic resonance imaging (MRI), and acute diseases such as gout [5] and scleroderma [49].

SIOT recommend IAHA once a week for 2–4 weeks, this treatment can be repeated after 12 months in patients without knee swelling or flares: low-molecular-weight hyaluronic acid is recommended for early/mild knee OA, while high-molecular-weight intra-articular hyaluronic acid is preferable in patient with severe OA who either are poor surgical candidates or must postpone total knee replacement [27, 28]

### Intra-articular injection treatments: second line (chronic therapy)

#### Growth factor/platelet-rich plasma (PRP) injection

PRP consists of a small volume of plasma with an increased concentration of autologous platelets and is prepared by blood centrifugation [50]. PRP injections are contraindicated in patients with haematologic blood dyscrasias with platelet dysfunction; septicemia or fever; cutaneous infections in the area to be injected; anaemia (haemoglobin less than 10 deciliters; malignancy, particularly with hematologic or bony involvement; and allergy to bovine products if bovine thrombus is to be used [5, 51].

Injection with PRP has the potential to improve pain and function for up to 1 year after treatment in patients with mild-to-moderate knee OA [52]. However, there is no consensus about PRP formulation in the literature, and most of the available society guidelines give inconclusive recommendations for use [3, 5]. However, given the increasing number of clinical studies [53] describing better clinical outcomes when compared with other conventional injectable treatments [50], this task force supports the use of growth factor and/or PRP injections in symptomatic knee osteoarthritis [54]. SIOT conditionally recommends PRP when other alternatives have been exhausted or have failed to provide satisfactory benefits.

### Intra-articular injection treatments: third line (only in clinical trials)

#### Mesenchymal stem cells (MSCs)

These cell-based products can be used in suspension after expansion in culture or enzymatic digestion. At present, their use is not recommended by scientific authorities [3, 4] because of the lack of standardisation in their preparation modalities [55], including sources of cells, processing methods, characterisation and administration technique [56]. Nevertheless, MSCs can be used in highly specialised centres, particularly in clinical trials, while they are weakly recommended in daily clinical practice as they are still being studied.

## Conclusions

For the conservative treatment of knee OA, SIOT strongly recommends focusing on lifestyle changes as the first step, particularly weight loss in combination with physical exercise and/or hydrotherapy. Patient self-management and education can be very useful, particularly if family members are involved, while aids such as canes, walking sticks, crutches and walkers are also extremely important to assist in walking. Balneotherapy represents a conservative treatment that may have beneficial effects on pain and stiffness and can be recommended.

Glucosamine and chondroitin are strongly recommended in clinical practice for chronic treatment, while NSAIDs (topical or oral formulations) are a better choice for acute symptoms, compared with acetaminophen. Specifically, SIOT recommends the use of oral non-selective NSAIDs (preferably with the addition of a PPI) or oral COX-2 selective inhibitor NSAIDs.

SIOT recommend opioid use only while patients are waiting for surgical treatment, if NSAIDs are ineffective against pain.

Considering intra-articular infiltrative therapy, the use of hyaluronic acid is recommended in cases of chronic knee OA in the absence of active acute disease, while the use of intra-articular injections of cortisone is effective and preferred for severe acute symptoms and represent the best treatment choice if their use is allowed.

**Fig. 2:**
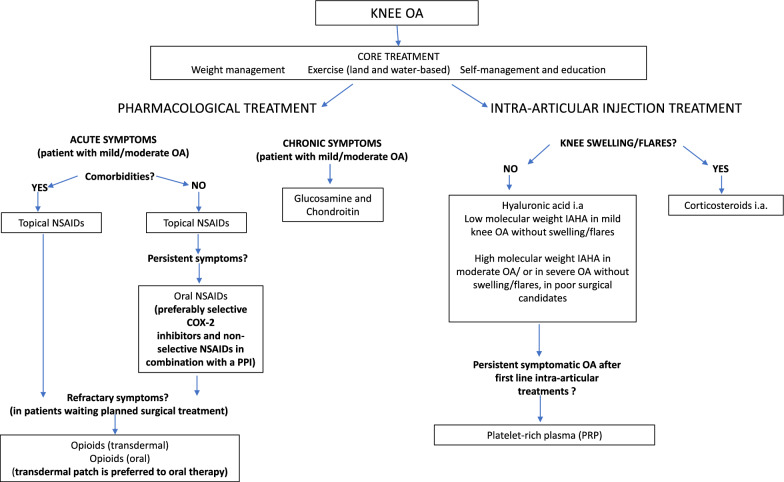
Flowchart of SIOT recommendations

The use of growth factor injections and/or PRP in symptomatic knee OA is only favoured in highly specialised centres, and only after intra-articular hyaluronic acid therapy has failed. The use of MSCs should also be restricted to highly specialised centres, particularly for clinical trials, while their use is not generally recommended in daily clinical practice as research into these cells is ongoing (Fig. 2).

## Data Availability

The data underlying this article are available in the article and in its online.
